# Bibliographical Notices

**Published:** 1844-10-01

**Authors:** 


### Periscope;

OR,

### CIRCUMSPECTIVE REVIEW.

"Ore trahit quodcunque potest, atque addit acervo."

### Notices of some New Works.

NARRATIVE OF A VOYAGE TO MADEIRA, TENERIFFE, MEDITERRANEAN, &c. By *W. R. Wilde,* M.R.I.A., &c. Octavo. Second Edition, enlarged. Dublin, 1844.

MR. WILDE, who is favourably known by his publication on Austria, and other works, was appointed medical attendant on a gentleman of fortune, voyaging in his own yacht, in pursuit of health and pleasure wherever their inclinations, wishes, or interests led them. A more delightful circuit could scarcely be made than our voyagers pursued. Although the Lexicographer never travelled farther himself than to Mull and Iona, he has stated it as his opinion that "he who wishes to see the world, must travel round the Shores of the Mediterranean." We wish we could follow Mr. Wilde in his comfortable and spruce Yacht to Egypt, Palestine, and most of the great Lions of Antiquity; but we dare not travel beyond the prescribed circle of medicine.

MADEIRA.

The impression on the minds of professional men is now all but universal, that Madeira offers the best chance of recovery from pulmonary complaints——when not too far gone; for then, Madeira, like every other southern locality, will only accelerate the fatal catastrophe. There are, generally, from two to four hundred English invalids who winter in Madeira, and many of them, who arrive late, have some difficulty in procuring accommodations. These are of three descriptions—furnished houses in or near the town, (amounting to nearly thirty,) each capable of containing a moderate family, and varying in expense from 60 to 200 pounds for the season. These it is necessary to bespeak before-hand. One female servant is worth half a dozen of males. Abundance of extremely honest Portuguese servants can always be procured. The second mode of accommodation is in family hotels, where small families may have separate apartments at 45 or 50 dollars per month per head. There are half a dozen or more of these hotels. The third consists of boarding-houses, with a *table-d'hôte,* and conducted much the same as in England. There are six or seven of these—all kept by English. The following extract contains useful information.

"In connexion with the subject of invalids, I here beg leave to offer some information upon the different modes of transit, derived from the most authentic sources, and brought down to the present time. During the last two seasons, the Peninsular Steam Navigation Company sent out one of their vessels, the Royal Tar, direct to the island. The prospectus states, that she 'will leave Southampton on the 18th of October; and after landing her goods and passengers at Madeira and Teneriffe, will proceed to Gibraltar, from whence she will make two trips to Madeira, leaving Gibraltar, on the first trip, about the 2nd, and on the second trip, about the 12th of November, to convey to the island such passengers as may be desirous of visiting the Peninsular ports (Vigo,

Oporto, Lisbon, Cadiz, and Gibraltar).' One of the same company's steam-ships will go out to Madeira in spring to bring home passengers. The latest and the amended prospectus of this company states, that passengers can be 'booked out in the fall and home in the spring of the year. Outwards—passengers have the option of two ways of proceeding, viz.: either by way of the Peninsula, in the Peninsular and Oriental Steam Navigation Company's Peninsular steam-ships, which start from Southampton for Gibraltar, every Saturday, at four P.M., calling at Vigo, Oporto, Lisbon, and Cadiz; at any, or all of which places, passengers will have the privilege of making such stay as they require, being taken from port to port by following steamers, without additional charge, and finally embarking at Gibraltar for Madeira, on board the Royal Tar, which vessel will make one or more trips for that purpose, between Gibraltar and Madeira, starting from the former place on or about the 4th of November, on her first trip—or, passengers may proceed to Madeira direct, by the company's steam-ship, the 'Royal Tar,' Captain G. Brooks, starting for Madeira, from Southampton, on the 18th of October next.

"Homewards—In the spring of next year, one of the Peninsular and Oriental Steam Navigation Company's steam-ships will make two, or as many more trips as may be necessary, between Madeira and Gibraltar, for the conveyance of passengers desirous of returning, *via* the Peninsula, and will return from Madeira direct for Southampton, the same as last spring.

"Rates of passage-money, which include a liberal table, with wines, spirits, &c., and also bedding, linen, and cabin furniture of every description—From Southampton to Madeira, first class, £30; second class, £21; children under ten years, half price; children under five, one third; children in arms (with the parent), free. Steward's fees, 15s. each first class passenger.

"Rates of passage out in the autumn, and home in the spring—Direct to and from Madeira, £55; by way of the Peninsula, £50. The Royal Tar carries an experienced surgeon. To secure passages, select berths, &c., apply at 44, Regent-street, Piccadilly; and the Peninsular and Oriental Steam Navigation Company's offices, 57, High-street, Southampton, and 51, St. Mary Axe, London.'

"The royal mail steamers for the West Indies proceed direct from Southampton to Madeira, leaving England the 2d and 17th of every month. This mode has only just commenced. All the steamers make the trip in from six to seven days.

"The Brazil packets still continue to touch regularly at Funchal. The East and West India ships also call there generally; but will only take passengers to or from the island when their berths to their final destination are not occupied; and this in England cannot be known till the last moment, so they are the least to be depended on.

"Three regular traders, the Grace Darling, Florence, and Vernon, fitted up with very comfortable accommodation for passengers, sail regularly once a fortnight. Many persons prefer them to the steamers; the passage is about twelve days on the average; the expense about £20. Besides these, there are always small Portuguese vessels trading to Lisbon and Gibraltar.

"Again, a steamer leaves Falmouth for Lisbon on Mondays, *regularly*. First cabin fare, £15; second cabin, £9. 10s., which includes table, &c. A steamer leaves Lisbon for Madeira every fortnight, and returns in a few days after she lands her passengers. Fares from Lisbon to Madeira (at present), first cabin, £10; second cabin, £7, including table; deck, £3. An English stewardess attends. The British mail contract-boats, which sail on Mondays from Falmouth, call at Vigo, Lisbon, Cadiz, and Gibraltar—touching at Oporto, and return by the same route, which is performed in eighteen or twenty days."

The heat of the Madeira Summer will be found too relaxing in general, and a return home in June will often be found useful.

Mr. Wilde is of opinion that the climate of Teneriffe is in no wise inferior to that of Madeira—indeed it is much drier. He thinks it admirably adapted to bronchial affections, with much expectoration. The towns are infinitely cleaner than Funchal—wheel-carriages can be more readily used—and great variety of climate can be easily obtained, by ascending some of the neighbouring hills. But it wants good accommodation!

We must now take leave of Mr. Wilde's highly amusing volume, which is pleasingly written, and full of information.

---

### Reports of the Pennsylvania Hospital for the Insane, for the Years 1841 and 43. By *Thomas S. Kirkbride,* M.D.

The Hospital for the Insane is an offset from the General Hospital, nearly two miles West of the City, and only began to receive patients in January, 1841. In reports of this kind there must always be a large portion of what is called "parish business"—but there is almost always something to interest the professional reader, however distant from the locality in question. The Pennsylvania Hospital appears to be well ventilated, heated by air, and supplied with good water. Being a recent construction, it has all the advantages of modern improvements. A considerable space round the buildings is dedicated to pleasure grounds for the recreation and health of the inmates. All classes—curable and incurable—are admissible, upon securing the payment of a reasonable sum for board—from three to five dollars per week.

In a large proportion of the recent cases some bodily disorder was cognizable, and the general health being restored, many of these recovered promptly from the mental aberration. In some, however, no bodily indisposition could be detected, and no indication appeared for medicine. All patients, unless for reasons to the contrary, are expected to take a warm bath at least once a week, for cleanliness; and there are all kinds of baths for medicinal purposes. The diet, except where bodily disease requires regulation, is ample—indeed we might say unlimited, both animal and vegetable.

Considering, as we do, that insanity is more or less connected with, or dependent on, corporeal disease or disorder, we apprehend that the above-mentioned scale of table pleasures is somewhat too liberal. The patients are treated in a mild and conciliatory manner, and "roughness or violence is never tolerated." Among employments and amusements, *out-door labour* stands pre-eminent. A farm, attached to the establishment, furnishes the means of exhibiting this valuable remedy. A workshop, has lately been constructed, which affords useful labour to particular classes, especially when the weather will not permit of outdoor employment. The Sabbath is strictly observed, and the influence of religion over the minds of the insane is remarkably illustrated on that day. Bodily coercion is not entirely relinquished in this Hospital.

"Simple seclusion in chambers properly secured, has been resorted to during the past year, in by far the greater number of cases that have appeared to require restraint of any kind. In others, leather wristbands, secured by a belt around the body, or mittens of the same material, or of canvass, have been employed, in rare cases, with a soft band about the ankles, and two patients have occasionally been kept on their beds with much advantage, by an apparatus also of leather, but admitting of much freedom of motion."

Among the numerous tables in this volume the following is a very interesting one.

Table—*Showing the supposed Causes of Insanity in* 176 *Patients.*

| | M. | F. | T. |
|---|---|---|---|
| Ill health of various kinds | 15 | 17 | 32 |
| Intemperance .. .. . | 16 | — | 16 |
| Loss of property, failures, &c. .. .. .. .. .. | 9 | 4 | 13 |
| Dread of Poverty .. .. | 2 | — | 2 |
| Disappointed Affections.. | 2 | 2 | 4 |
| Intense Study.. .. .. | 4 | — | 4 |
| Domestic Difficulties .. | 1 | 3 | 4 |
| Fright .. .. .. .. .. | 2 | — | 2 |
| Grief, loss of Friends, &c. .. .. .. .. .. | 2 | 10 | 12 |
| Intense application to business .. .. . | 1 | — | 1 |
| Religious Excitement . | 3 | 5 | 8 |
| Want of Employment .. | 6 | — | 6 |

| | M. | F. | T. |
|---|---|---|---|
| Celibacy .. .. .. .. .. | 1 | — | 1 |
| Mortified Pride .. .. | — | 1 | 1 |
| Anxiety for Wealth .. | 1 | — | 1 |
| Use of Opium .. .. | — | 1 | 1 |
| Use of Tobacco .. .. | 1 | — | 1 |
| Child-birth .. . .. | — | 5 | 5 |
| Uncontrolled Passion .. | 1 | 1 | 2 |
| Tight Lacing .. .. .. | — | 1 | 1 |
| Injuries of the Head .. | 2 | 1 | 3 |
| Masturbation .. .. .. | 2 | — | 2 |
| Mental Anxiety .. .. | 2 | 1 | 3 |
| Exposure to Cold .. .. | 1 | — | 1 |
| Unascertained.. .. .. | 29 | 21 | 50 |
| | 103 | 73 | 176 |

Of sixty-one patients who had been discharged, or who died in one year, we find that 30 were cured—that 5 were much improved—that 6 remained stationary—and that 9 died. Among the nine deaths, one was a case of typhus fever, the delirium of which was mistaken for insanity—the second was from dysentery—the third from chronic inflammation of the bowels and stomach—the fourth from phthisis—the fifth from meningitis—the sixth from hydrothorax—the seventh from erysipelas—eighth and ninth from suicide.

From a Second Report, for the year 1843, we shall make a few short extracts.

"*Restraint.*—Although the means heretofore detailed, and the aid of a vigilant and efficient corps of assistants, have enabled a large number of the patients to enjoy the privileges which I have mentioned, almost from their first entrance; it is not to be concealed, that we always have in our family, some with that unfortunate temperament, that blackens the fairest scenes,—distorts the purest motives, and misconstrues the kindest actions; and that many require some more decided restraint, until the violence of their attack has subsided."

And again—

"Restraint of some kind is one of the means for treating insanity, which, to a greater or less extent, will always be required; but all that is proper, does not necessarily imply the use of apparatus, to which the term has generally, but with great impropriety, been restricted. The amount of restraint at all justifiable is still so little understood in many sections of country, that the subject cannot be too frequently referred to, in the reports from Hospitals for the Insane.

"I have stated in a previous report, and have seen no reason to change that opinion, that it is quite possible to dispense with all restraining apparatus, in a well-constructed Hospital, properly supplied with an efficient corps of attendants; but that, under certain circumstances, the use of the milder means may be a comfort to the patient, and much less annoying, than constant struggles with, or even the presence of, the best instructed and most humane attendant. These cases, however, are not common. Having no exclusive views on this subject, apparatus has been used, here, in every case, where I saw a rational indication for its employment.

"The only means of the kind we ever employ in this Hospital, consist of an apparatus which effectually retains a patient on his bed, while admitting of considerable motion; leather mittens or wristbands, secured by a belt around the body; and a strong cloth jacket, with continuous sleeves."

We are thoroughly convinced that a mild *mechanical restraint*, in obstinate cases, is less irritating to the patient than the personal efforts of keepers to effect the same object.

### Researches into the Physical History of Mankind. By *J. C. Pritchard,* M.D. &c. &c. Vol. the Fourth, 1844.

The goal of this stupendous work is within the view of its author. One more volume will complete an undertaking that has no parallel in the English or in any other language. No similar work will be attempted for ages to come. No author could hope to equal, much less surpass, the researches of Dr. Pritchard. An imitation could only be a series of plagiarisms, or a tissue of errors. To attempt an analysis of a work of this kind would be fruitless. Besides, the investigation is equally interesting to every class of readers, though not strictly adapted for extensive notice in a journal of practical medicine. There are certain portions of this volume, however, which are peculiarly attractive to the medical reader, and from these we shall select a few characteristics.

### 1. *Physical Characters of the Persian Races.*

Sir J. Chardin, in particular, asserts that the Persians are an ugly ill-favoured race; and he ascribes this to their non-intermarriage with the neighbouring Circassian and Georgian females. It is true that the Southern Persians are darker coloured than the Northerns; but this may be accounted for by climate and other causes. The numerous sculptures of the Medes and Persians on the walls of Istakar and Hamadan, prove that the Persians *were* amongst the most beautiful of the human race. Actual observation in modern times, has proved that their descendants have not sensibly degenerated, in this respect, from their ancestors. They have not, indeed, the fine intellectual countenances of the Greeks; but they display features of the Indo-European style, with long faces and high foreheads.

### 2. *Physical Characters of the Native Indians.*

This race embraces such a multitude of genera and species, that it is next to impossible to sketch any thing like a general character. It has been asserted that the Aborigines of Hindostan were a tribe of Negroes with woolly hair and the African features. Dr. Pritchard once leant to this hypothesis, but has, long since, abandoned it. As far back as historical records reach, the Indians had straight or lank hair, and features very unlike the Negroes. Herodotus describes them accurately, as divided into castes as at the present day. Dionysius, the Geographer, is much more particular as to the physical character of this ancient people.

"The inhabitants are swart, and in their locks betray the tint of the black hyacinth."

In general the Hindoos have small foreheads—the face thinner and more meagre than Europeans, and they are much inferior to them in strength. They are lean, feeble, and incapable of supporting hard labour and fatigue. So says Dubois, but he is mistaken. The Hindoos will bear fatigue in their own country which would overwhelm and destroy the European. In Bengal the greater number are below the middle stature; but in the upper provinces it is very different. Some of the Hindoos are as black as the Negroes, which surprised Bishop Heber much. But, in fact, the shades are endless, from nearly white to jet black. Their gesture and garb smack of the maidenly or even the effeminate; with sly countenance, yet smiling out a glozed and bashful familiarity.

3. *The Chinese.*—This wonderful people, now more interesting to us than ever, form a peculiar group of nations, who boast a civilization of their own, a development and cultivation of intellect—a growth of arts, knowledge, and religion, independent and indigenous, deriving nothing from the aid of foreigners, either in the earlier or later ages of the world. Almost as separate from the countries of Central Asia as were the civilized nations of Peru and Anahuac, the Chinese lived under the patriarchal rule of powerful Emperors long before their name or country were heard of in Europe. For centuries afterwards, they were only known as possessors of a country which produced the most splendid manufactures of the East. The poetical Greek Geographer describes them as—

> ——————— "barbarous tribes who use
> Nor oxen hides, nor wool of fatted ewes;
> They weave sweet flow'rets of the desert earth
> Of finest texture and of richest worth—
> Robes bright of hue as flow'rs that deck the mead,
> Of finer texture than the spider's thread."

The physical character of High Asia seems, in the Chinese, to be softened down and modified into the European type. Customs, habits, laws, and language combine to amalgamate the various tribes of this extensive empire. Dr. P. cannot venture to consider the Chinese, Japanese, and Koreans as belonging to one race or family, because their languages are not referrible to one original. "Yet they are, if we regard their physical characters, one sort or stock of people. No human races bear a stronger resemblance to each other than these three people. They all have the same physical type." Whether the same type was originally impressed upon them, or resulted from long-continued influence of external agency, will never now be ascertained. In either case, the effect is the same.

LINNÆUS has characterized the Chinaman as—"Homo monstrosus, macrocephalus Chinensis." This is a monstrous exaggeration. The small eye, elliptical next the nose—high cheek bones—pointed chin, are prominent features in the Chinese countenance, while the shaving off the hair gives to the head the shape of an inverted cone. The natural colour of the skin is between a fair and a dark complexion—or that of a European brunette, becoming brown in the labouring classes from exposure to the sun. Many females in China, among the better classes, would pass for beauties in Europe.

They are of the middle stature—small hands and feet—face flat—nose small—eyes prominent—hair black and stiff—scanty whiskers and beard. The women, in general, are far inferior to those of Georgia, and some other parts of Asia.

But we must conclude with these few specimens of Dr. Pritchard's highly interesting volume. When he has completed the fifth tome, he may well exclaim—

"Exegi monumentum aere perennius."

---

### ON THE OCCURRENCE OF SARCINA VENTRICULI ALONG WITH ACETIC, LACTIC, AND CARBONIC ACIDS IN WATER-BRASH. By *George Wilson*, M.D. Lecturer, &c. Edinburgh.

IN this paper the attention of pathologists is directed to some curious phenomena accompanying certain forms of morbid digestion in the human subject. Of these the most remarkable is the appearance of a microscopic cryptogamic plant *(Sarcina Ventriculi)*, and of acetic, lactic, and carbonic acids, in a liquid ejected from the stomach. Mr. Goodsir published the first case of these in the Edin. Med. and Surgical Journal for April, 1842. In fact, five cases of sarcina have occurred very recently and within a very short space of time; which renders it not improbable that the development of this organism is no uncommon accompaniment of certain derangements of the digestive functions. Dr. Wilson here presents us with the more important particulars of the cases already seen, with some observations on the manner in which the chemical and microscopical phenomena are related to each other, and to the state of the system which attends their manifestation. The first case occurred in a young man of 19, who had been suffering for months from what he supposed to be water-brash. He stated that it attacked him on awakening in the morning with a feeling of distention of the stomach; that without any effort of vomiting, a quantity of fluid, varying in volume from two thirds to a whole wash-hand basinful, passed up from his stomach; that after this he was quite relieved, and experienced no further inconvenience till the evening of the same day, when, without decided distention, sounds as of a fluid boiling or bubbling, and proceeding from the region of the stomach, were perceptible to himself, and to those around him; that he slept well enough, but was generally attacked next morning—the fluid ejected by this patient "smelt like fermenting worts, with a faint acid odour. It appeared, after standing for a few hours, moderately transparent, and of a light brown colour. It deposited in the bottom of the basin a quantity of a ropy matter of a granular appearance, and on the surface was a mass of froth like the head of a pot of porter." On examining the fluid with the microscope, the sarcina was at once detected and was found to present the following characters. In every instance the organisms presented themselves in the form of square or slightly oblong transparent plates, of a pale yellow or brown colour, and varying in size from the 800th to the 1000th of an inch. They were made up of cells, the walls of which appeared rigid, and could be perceived passing from one flat surface to another as dissepiments. These dissepiments, as well as the transparent spaces, were from compression of contiguity rectilinear, and all the angles right angles; but the bounding cells bulged somewhat irregularly on the edges of the organism by reason of freedom from pressure. These circumstances gave the whole organism the appearance of a wool-pack, or of a soft bundle bound with cord, crossing it four times at right angles, and at equal distances. From these very striking peculiarities of form, Mr. Goodsir has proposed for it the generic name of SARCINA.* The ejected fluid was thick and viscid; on standing, it deposited a large quantity of ropy matter mixed with portions of undigested food, and when filtered through paper had a pale brownish yellow colour and was quite transparent—it contained much animal matter in solution, and gave a copious precipitate with infusion of galls—it also precipitated nitrate of silver densely, and when evaporated to dryness, and exposed to a full red heat in a platina crucible, left an ash containing much chloride of soda—it reddened litmus and effervesced sharply with alkaline carbonates. It was found also by neutralizing the fluid, after distillation, with lime water, and decomposing the salt thus formed by means of sulphuric acid, that it contained acetic acid. After distilling all the acetic acid, it still reddened litmus; this was owing partly to the presence of a little free muriatic acid, but chiefly to lactic acid. The most remarkable feature in this case was the large quantity of acetic acid present.

Mr. Bell's patient was a girl aged thirteen, who was subject, after her meals, particularly dinner, to enormous distention of the abdomen, accompanied by a gurgling noise and fetid eructations. From this she was relieved by vomiting "a thinnish, moderately transparent fluid, with a thicker and more tenacious portion, like gruel, at the bottom of the vessel—the surface sometimes covered with froth." In this, Mr. Goodsir found the sarcina in great abundance. The liquid from Mr. Bell's patient was found to correspond with that from the first patient. The frothy appearance depended on the presence of carbonic acid, which was proved by experiment to be present in the liquid ejected by the second patient. The frothy appearance in the liquid given by the first patient may fairly be presumed to depend on the same cause. Thus, then, the liquids from both patients contained, besides undigested food, salts and animal matter, much acetic,

---

* *Sarcina*, a wool-pack.

lactic, and carbonic acid, and in one case a little muriatic acid, the quantity of carbonic acid being decidedly abnormal, as was also that of the acetic and lactic acids.

The ejected liquid, it may be well to notice, must have consisted partly of secretions from the stomach, partly of products of digestion—the bulk of the liquid being secreted by the stomach, and the acids being derived from the food. We already alluded to the resemblance between the ejected matter and a fermenting liquid; in fact it bore a striking resemblance to a vegetable juice undergoing lactic fermentation. The sarcina too might be considered as the analogue of the microscopic plants which are found to accompany the fermentation of infusion of malt and the juices of different fruits—hence the disease was supposed to result in great part from the food undergoing within the stomach a peculiar fermentation; three different views, in fact, might be entertained concerning it.

1. The disease might be regarded as depending on a simple or spontaneous fermentation of the food. 2. The *sarcina* might be considered as the *causa morbi*. 3. The stomach might be looked on as furnishing the ferment, and the sarcina as an accompaniment only of the fermentation excited. The last view seems the most probable. According to recent researches, the performance of digestion depends on the presence in the stomach of two different substances,—the one, free muriatic acid, the other, an animal matter called *pepsine*. Infusion of pepsine has no solvent action on alimentary substances, but its addition, even in minute quantity, confers this property on water acidulated with muriatic acid. How the pepsine acts in conferring this power, is a disputed point. Liebig represents the pepsine* as an efficient cause of alteration in the food, solely because its own particles are in a state of decomposition or transformation. The pepsine bears the same relation to the food, whose digestion it effects, that yeast does to the sugar it ferments. The transforming action of pepsine is limited in the normal state to rendering the insoluble portions of the food soluble in the gastric juice. Now all the azotised bodies, as diastase, yeast, &c. which act as ferments, and among which pepsine may be ranked, pass through several stages of decomposition whilst performing this function, and modify the non-azotised bodies on which they act, according to the state of decomposition in which they themselves are. From these considerations it appears that, within certain limits, if the mode of decomposition of the ferment alters, the mode of decomposition of the fermented body must alter also. Thus, if normal digestion be the result of a constant decomposition of the pepsine in one way, it must be liable to be rendered abnormal by the pepsine decomposing in a different way. The cases already cited seem to be derangements of digestion of this kind. Normal digestion is here looked on as a fermentation, and pepsine as a ferment. The carbonic, acetic and lactic acids, found in such abundance in the ejected fluids, were products of the fermentation or transformation of the food, determined by its contact with the modified pepsine.—*Northern Journal of Medicine.*

---

### Letter to the Home Secretary of State. By *J. Perceval*, Esq.

This epistle, with a score or more of documents and letters, appears to be a somewhat romantic, if not quixotic campaign against the Metropolitan Commissioners of Lunacy, on account of their "unjust and pettifogging conduct towards a gentleman lately under their surveillance." Mr. Perceval appears to have waged war, not only against the Commissioners, but against the Lord Chancellor,

---

* Liebig does not actually employ the term *pepsine*.

nay, against the Law itself! The author is a bold man, and has certainly taken the bull by the horns. Of Mr. Perceval's mode of warfare and the kind of weapons which he wields, the following passage will convey some idea.

"Dr. Southey and Dr. Bright, many years ago, I came in contact with, on which occasion I do not think that their conduct was just or straightforward. Of Dr. Bright I can say that I never saw a countenance that shocked me more strongly, as stamped with the expression of Lunacy." Dr. Southey has not fared much better under the hands of Mr. Perceval. "Dr. Southey has too much of the *suaviter in modo* to be depended upon for the strict discharge of his duty, where rank and power are arrayed against poverty and helplessness."—p. 6. Surely Mr. Perceval cannot expect that any great attention can be paid to animadversions mingled with such personal invectives as the above. In respect to his protegée—the Man in mask or in blank—whose incarceration in mad-houses Mr. P. bewails and labours to avenge, we very much fear that his recriminations and complaints are greatly exaggerated by the keenness of his own feelings and the dolorous reminiscences that rise through a haze in his mind's eye. It is not often, indeed, that we hear of liberated maniacs lauding their former keepers, or praising the diet and discipline of the asylum. We shall glance, however, at some of the grievances which the Man in blank sets forth in a letter to the Commissioners of Lunacy. For sixty-five nights he was annoyed by "a colony of rats under the floor." "No pen or tongue can describe the misery and agony of my feelings; nor the horrors of those sleepless nights passed in a gallery filled with twenty to thirty pauper maniacs, amidst their cries, and moanings, and howlings, and blasphemings, &c. &c." Now, we really think that the "Colony" under this Pandemonium had no small reason to complain of the annoyance which they sustained from their superiors in the gallery. The complainant states that, at the Establishment in question, which shall be nameless, there was a paucity of keepers, and that he himself was sometimes obliged to assist in applying the strait-waistcoat round his refractory messmates. The want of sufficient firing is loudly complained of, and the cheese was such that, had the rats known the quality of that which they were in seach of, they would have directed their forays into another channel.

We are happy, however, to know that, since the Man in blank set forth his grievances, great reforms have taken place, moral and physical, in Lunatic Asylums, and if Mr. Perceval, by his former exertions and remonstrances, has contributed to these ameliorations, he may rest contented with the laurels he has gained.

---

### On the Physiological and Medicinal Properties of Bromine and its Compounds; also, on the Analogies between the Physiological and Medicinal Properties of these Bodies, and those of Chlorine and Iodine, with their Correspondent Compounds. By *R. M. Glover*, M.D. Edin. (Notice from the Edinburgh Med. and Surg. Journal, No. 152.)

The subject of this Paper, which was proposed for the Harveian Prize Essay of 1842, is extremely interesting, as well for the results more immediately arising from its consideration, as for its contributing to the solution of a problem of the highest interest, viz. the extent to which the chemical analogies of bodies are accompanied by analogy of action on the living system. The idea of a relation between the chemical and physiological properties of bodies, similar to that which exists between the external forms of many plants and the action of the same plants on the animal economy, has been frequently suggested. The author first alludes to the remarkable analogies of physical character in chlorine, bromine, and iodine, which may in general be traced in a modified form throughout the long series of compounds. Thus, at ordinary temperatures, chlorine is a light green coloured gas; bromine a liquid of a splendid red colour; iodine a solid with an intense shade of violet. And the volatility of their respective compounds in general follows in each case the rule of the element. Hydrochloric acid is the most volatile of the three hydracids, and hydriodic acid the most fixed. The chloride of potassium is volatile at a brown red heat, while the iodide requires a much more elevated temperament. Chloride of cyanogen is gaseous at ordinary temperatures, when the bromide and iodide of the same substance are solid. He next adverts to the relation which is found to subsist between the specific heats of these bodies, and also between their chemical equivalents. From the chemical facts ascertained by investigating the several habitudes of these bodies, the author concludes, that bromine is more allied in chemical properties to chlorine than to iodine. The following is the arrangement observed by the author in the composition of his Essay.—1st. He considers the Physiological Properties of Bromine and its Compounds—the Hydrobromic Acid, Bromides of Potassium, Sodium, Magnesium, Barium, Zinc, Iron, Mercury, Cyanogen and Olefiant Gas, with Bromiform, and the Analogies which they offer with Chlorine and Iodine, and and their correspondent compounds;—2nd. The Medicinal part of the Enquiry; 3rd. General Conclusions.

PHYSIOLOGICAL PROPERTIES OF BROMINE AND ITS COMPOUNDS.

All substances capable of exerting a chemical action on component parts of the tissues of organized beings, must be irritant, the phenomena which appear in such cases being partly of a re-active character. The exact nature of the action of bromine is as yet obscure. But little is known on the subject, save the mere fact of the coagulation of albumen by bromine. Considerable doubt exists with respect to the action of all corrosive substances on the animal tissues. Mulder describes a series of regular atomic compounds formed by the action of acids on albumen, such as the sulpho-proteic acid. Looking at the analogy of chlorine, we have the chloro-proteic of Mulder.

According to Balard, bromine readily attacks organic matters, such as wood, linen, and especially the skin, which it colours strongly yellow; the tint being less intense than that given by iodine, and disappearing like it at the end of some time. In these circumstances, according to Berzelius, water is decomposed, the oxygen is carried to the organic substance, and the hydrogen forms hydrobromic acid with the bromine. It is not easy to see, observes the author, how, on the coagulation of albumen by bromine, a bromoproteic acid could be thus the sole resulting compound. When white of egg is coagulated by bromine, either pure or in solution in water, the colour of the element is lost. The coagulum is soluble in solution of caustic potash with or without the aid of heat. From this solution we can readily obtain evidence of the presence of bromine. If, without dissolving the coagulum, we wash it on a filter with distilled water, the liquid that passes, assumes a faint blue tinge, and is a solution of a small quantity of albumen in dilute hydrobromic acid. Conceiving the existence of a chloroproteic acid to be altogether hypothetical, the author prefers explaining the changes described, by supposing that compounds of albumen, with bromine and hydrobromic acids, exist in the clot, the albumen serving as a feeble base; but when the clot is washed, these acids are decomposed, and a weak solution of albumen in hydrobromic acid passes the filter.

The action of hydrobromic acid on albumen presents a most beautiful analogy with that of hydrochloric acid. When white of egg is put into concentrated hydrobromic acid, it is at first coagulated, but by repeated agitation in excess of the acid it is dissolved, and with more facility by the aid of heat. The result is the formation of a deep-coloured solution, not of a deep indigo blue tint, as in the case of hydrochloric acid, but of a brownish purple hue. When bromine in water is placed in contact with fibrin, the latter substance is converted into a bluish gelatinous-looking body. The same circumstances are observed of fibrin in this state as of albumen coagulated by bromine. A solution of colouring matter of the blood is coagulated by bromine, the hue of the mixture becomes olive-green, and ultimately gray, if excess of the element be employed. And it may not be without interest to observe, that, on washing the coagulum, the iron of the blood is removed, and yet, when the washings cease to give evidence of containing iron, a large excess of bromine having been employed, the precipitate left in the filter, on being boiled with solution of caustic potass or soda, will give a colour nearly resembling that of the blood. This fact may be adduced in connexion with the argument of M. Scherer, who has sought to prove that the colour of the blood is not entirely dependent on its iron.

Bromine exerts an action on the fatty principles of the tissues, and appears to be transformed into hydrobromic acid at their expense. The compounds formed are probably analogous to those so formed by chlorine. With respect to the physiological properties of bromine and its compounds, sufficient evidence has been adduced by former investigators to shew that the primary action of bromine, introduced into the stomach, or into the circulation, bears considerable resemblance to that of chlorine or iodine, in so far as either of these bodies can be similarly administered. The following facts may be stated with advantage, as the results of former investigations. M. Balard, the discoverer of bromine, observed that a drop of it placed on the back of a small bird soon produced death. Barthez found ten or twelve grains of bromine dissolved in a sufficient quantity of water to occasion death when injected into the jugular of a dog. A single fit of tetanus only preceded death. Sometimes, in such an experiment, death did not take place, but there were restlessness, dilated pupil, frequency of pulse, and sneezing. Blood-letting after the commencement of the symptoms favoured recovery in such cases. The author gives passages from Devergie descriptive of the symptoms occasioned by the introduction of bromine into the stomach of animals. Introduced into the stomach of a dog in a dose varying from 30 to 40 drops it occasions death, giving rise to:—nausea, attempts to vomit, vomiting, acceleration of the respiration and circulation, prostration continuing till death—the pathological alterations being, injection of the villous coat of the intestinal canal, with corrugation of the gastric portion of this membrane; now and then grayish superficial ulcers, and in some cases softening. These effects have much resemblance to such as have been observed to arise from the similar administration of chlorine and iodine. The author here details a series of experiments instituted for the purpose of ascertaining the action of bromine on the system. The first was with a gold fish placed in a mixture of one part of saturated solution of bromine and two parts of water—its whole surface was immediately corroded, and life was extinct in less than a minute. Four drops of bromine were placed on the bill of a pigeon—the bill was corroded—violent excitement followed by an apathetic state—death in two days from corrosion and irritation of the air-passages caused by inhaling the fumes of the bromine. Four drops of bromine were introduced into the external jugular of a strong male rabbit—he immediately shrieked; respiration laborious—pupils dilated—heart's action violent, but ceased after a few irregular beats—two or three convulsive struggles; the animal was dead in 70 seconds from the commencement of the operation. On opening the chest, the irritability of the heart was found to be destroyed—the substance of this organ was corroded near the apex—several marks of corrosion in the lung. Blood in the right ventricle and pulmonary artery coagulated. Similar experiments were instituted with the same results on a terrier bitch and a strong fox hound. The post-mortem appearances in the two latter subjects were—congestion of lungs with several apoplectic-like spots—bronchi contained frothy blood. Stomach contained about half a pint of grumous blood—its mucous coat was much injected and presented several large ecchymoses—rectum vascular. From the results of these experiments we may conclude that bromine introduced directly into the circulation appears to exert a corrosive and irritant action on any organ to which it may be directed—in the experiment on the fox-hound, it was found to produce death by coagulating the blood and so obstructing the circulation. The sudden stoppage to the circulation through the left side of the heart, and consequent compression of the nervous centres, may account for the nervous symptoms which occur in such cases.

Two series of effects are produced by the introduction of bromine in large doses into the stomach,—1st, from the action due to volatilized bromine getting into the fauces and air-passages, and so into the lungs; and 2d, from the corrosive and irritant action on the stomach and intestines. Another series of effects might be due to the entrance of bromine into the circulation. To the first series might be referred the coryza, sneezing, salivation, and affection of the respiration. The solution of a part of the stomach, as observed in one of the experiments, may be explained by the conversion of the bromine still tarrying in the stomach into hydrobromic acid. The absorption of hydrobromic acid is corroborated by numerous analogical facts. Chlorine and sulphuretted hydrogen pass into the urine, also arsenious acid. The hydracids of chlorine, bromine, and iodine are peculiarly likely to be absorbed, since the two former form soluble compounds with albumen, and we may assume as much of the other.

The author here details the observations of M. Fournet on the physiological effects of bromine, which were manifested on some patients of his, affected by chronic arthritis. The remedy was given suspended in mucilage. The dose was at first two drops, but was raised by two drops daily, until it amounted to sixty drops in the 24 hours. The quantity of mucilage employed was always four ounces. Two drops thus taken occasioned only a hot sensation in the back of the palate. In a little stronger dose the patient experienced in a quarter of an hour itchings in the hands and feet, and shocks in the feet and near the knees. After a quarter of an hour there were borborygmus and colic. As the dose became stronger, there was a sensation of heat in the chest, accompanied with attempts to vomit, but without any vomiting. At first, a peculiar sensation of weakness and fatigue in the chest accompanied these first efforts, but these symptoms, from habit, soon disappeared. The patient who had the itchings in the fingers was always the most sensible to the effects of bromine. Tonic and diuretic effects have been experienced from bromine. That it acts on the nervous system is evident from its occasioning dilated pupil and stupor.

*Hydrobromic Acid.*—Several experiments were instituted to ascertain the physiological properties of this acid, and it was found to be less powerfully irritant and corrosive than the element.

The author next details the results of some experiments undertaken to ascertain *the Properties of the Bromides of Potassium and Sodium.* These experiments enable us to compare the operation of the bromide with that of the iodide of potassium. According to Mr. Blake, the salts of potash, when injected into the veins, destroy the irritability of the heart, and when introduced into the arteries, obstruct the circulation through the systemic capillaries. The experiments made by the author prove both these properties to be possessed by bromide of potassium. The bromide of potassium is found to be less powerful than the iodide of the same base. The bromide of sodium appears to bear a close resemblance to common salt in physiological properties; it bears a great resemblance to it in taste. The *bromide* and *iodide of barium* were found by experiment to possess the physiological properties of the class of salts to which they belong, more especially resembling the chloride of the same base. From the results of experiments made with *bromide of magnesium,* there seems reason for concluding that this compound of magnesium participates in the general physiological properties of the magnesian salts. It is less active than the bromide of zinc, still the effects produced by it very much resemble those caused by the zinc salt.

It was ascertained by experiment that the *bromide of zinc*, when thrown into the venous system, puts a stop to the motions of the heart, without, however, totally destroying the irritability of the organ; and that, when this salt is introduced, so as to pass through the systemic capillaries before reaching the heart, it obstructs them temporarily, but exerts its deleterious influence on the central organ of the circulation when it arrives there in quantity. The experiments instituted with *bromide of iron* establish the analogy of it with other salts of the same base, and more especially with the iodide. When injected into the veins, it appears to obstruct the pulmonary circulation and weaken the heart's action; when these effects have in some degree subsided, a powerful secondary action on the intestinal canal becomes apparent.

The *Bromide* and *Sub-bromide of Mercury* bear the most remarkable resemblance in chemical properties to the chloride and sub-chloride, to which also their physiological affinity is strict. With respect to the *Bromide of Cyanogen*, it appears, from the results of numerous experiments instituted by the author, to possess two kinds of action; one on the spinal cord—perhaps primarily on the sympathetic system, closely resembling the primary effect of prussic acid. The secondary or irritant action far exceeds in intensity that of prussic acid. This body has little corrosive power, it coagulates white of egg but slowly, and is as nearly as possible a pure irritant in its secondary action.

We shall now pass on to consider the—

### Medicinal Properties of Bromine and its Compounds.

Bromine was first used as a medicinal agent by M. Pourché. Before this, Desorgues, calling himself a *simple magistrate*, had written to the Academy of Medicine, proposing the employment of bromide of mercury in the treatment of syphilis.

M. Bonnet, in a paper published in the *Bulletin General de Therapeutique*, July 1837, in detailing all that had been then observed in France with respect to the medicinal uses of bromine and its compounds, refers to the researches of M. Pourché. In a case of scrofulous enlargement of the glands of the neck in a woman of 22 years of age, who had been affected for seven years, a cure was effected in three months by the external and internal use of bromine. At first, six drops dissolved in three ounces of water were given in the day, in three doses. Next day ten drops were given. In ten days the dose was increased to fourteen drops daily, and at last to thirty drops in the same quantity of water. Cataplasms, moistened with solution of bromine, were applied to the swellings. The same physician had great success in the treatment of scrofula by the internal and external use of the hydrobromate of potass. M. Bonnet attributes great benefit to the bromide and sub-bromide of mercury, as constitutional remedies in comparison with corrosive sublimate and calomel, the former having, he says, less action on the salivary glands, and more on the urinary secretion. Magendie employs bromine and its preparations in scrofula, amenorrhœa, and in hypertrophy of the ventricles. Dr. Williams has used the bromide of potassium with success in cases of enlarged spleen. The first case which he gives is that of a boy, aged 14, in St. Thomas' Hospital. Both the liver and spleen were enormously enlarged. Their edge was hard, and substance unyielding. Abdomen contained much fluid—countenance sallow and emaciated—legs dropsical—belly protuberant—the ordinary remedies were tried in vain; at length the iodide of mercury was used—this removed the dropsy, but the liver and spleen remained enlarged. The patient now commenced the use of the bromide of potassium, one grain thrice a day, which dose was gradually increased to four grains. After using it for nearly two months he became slightly jaundiced—the jaundice was removed by the sulphate of magnesia, and in a few weeks the bromide was resumed; when, after its continued use for some time, he improved gradually, and was dismissed with the liver and spleen considerably diminished.

The author now gives a summary of the results obtained in his own practice. In a case of obstinate eczema of the legs and arms in a married woman, aged 40, of strumous habits, a saturated solution of bromine mixed with water until it ceased to give pain, and applied by means of lint and oil-skin, caused a decided improvement to take place. In two months the patient was cured. The second case mentioned by him was one of specific ulcers of the legs of long standing. After various metallic washes had been applied to the ulcers without any benefit, they were treated with a strong etherial solution of bromine, which acted as a caustic, and lint steeped in a saturated solution of bromine placed over them, covered with oil-skin. This application, which was repeated next day, produced pain and intense redness, and was discontinued. The ulcers healed rapidly afterwards, and cicatrization took place. The third case was one of carbuncle, which, after resisting the hydriodate of potash, used internally and externally, was cured by the external use of bromine—forty minims to the pint of water—in between six and seven weeks. In another case of scrofulous ulcer of the leg, in a boy aged 12, with a similar ulcer on the back of the right wrist, about the size of half-a-crown, in which tonics and hydriodate of potash had been used without success, the lotion of bromine, in the dose of 40 minims to the pint of water, was applied externally three times a day by means of lint and oil-skin, while the bromide of potassium was given internally. In six weeks the ulcer on the leg was nearly healed—that on the wrist continued open. He was then made an out-patient, and died in the course of the winter of diabetes. Several other cases are given by the author, where the bromides were employed with varying success.

From all the author has witnessed with respect to the real therapeutic power of bromine and its compounds, he is disposed to recommend the external use of bromine in scaly dartrous affections of an inveterate character, in specific and malignant ulcers, where there is defective action. Oil-skin should be employed to cover the lint in which the bromine is dipped, to prevent evaporation. Internally the use of bromine must necessarily be very limited.

We have now given a concise analysis of this interesting paper, which does great credit to Dr. Glover, and proves him to be an industrious and judicious experimenter in a department of medical science, so very important, because so decidedly practical.

---

THE AMERICAN JOURNAL OF INSANITY. Edited by the Officers of the Lunatic Asylum in UTICA. No. 1. July, 1844.

BROTHER JONATHAN is, assuredly, "going a-head" in physic as well as in commerce, and all the various branches of art, science, and literature. Free, or at least democratic, institutions, have a general tendency to liberate the mind from the shackles and forms imposed on it by despotic governments; as may be seen in a comparison of China with Great Britain. But, as America is still more democratic than England, so, in the former, there is greater propensity to spurn the boundaries within which the current of thought, invention, and speculation runs in the "Old World." The "JOURNAL of INSANITY" conveys a new idea; and the wonder is that it never struck the encephalon of John Bull, who is not a little prone to this terrible malady, and who expends many millions annually on institutions for its reception and treatment.*

* There is a Journal of Insanity lately established in France, (Annales Medico-Psychologiques,) by Drs. Baillarger, Cerise, and Louget.—*Ed.*

The Asylum in UTICA is a recent and state establishment, and among the Board of Managers we recognize some good names, such as Dr. Theodore R. Beck, Dr. Coventry, &c. &c. The resident officers of the Asylum include Drs. Brigham and Buttolph. The following sentence is gratifying :—"*It is believed that the proper discipline can be established among the maniacs without the use of the whip.*"

The second article in this new contemporary is entitled—"*Insanity illustrated by the Histories of Distinguished Men, and by the Writings of Poets and Novelists.*" Our countryman, COWPER, is the first that comes on the stage, since he was not only insane, but a poet, and has given us portraits of himself as well as of others in that melancholy predicament. His own feelings, however, can hardly be depended upon when his intellects were deranged ; but his delineations of CRAZY KATE, shew the poet and the observer in every line.

"She heard the doleful tidings of his death,
And never smiled again ! And now she roams
The dreary waste—there spends the live-long day.
She begs an idle pin of all she meets,
And hoards them in her sleeve."

It is curious that this begging of a pin is by no means uncommon among the insane.

In Childe Harold, Byron, no doubt, refers to his own case.

——— "I have thought
Too long and darkly, till my brain became,
In its own eddy boiling and o'er wrought,
A whirling gulf of phantasy and flame ;
And thus untaught in youth my heart to tame,
My springs of life were poisoned."

Johnson has described monomania in the person of the Astronomer in Rasselas, who thought that he ruled the planetary system. Every one now acknowledges the close connexion between genius and insanity, of which we have more than one example among the Lions of our own days. But we cannot say that stupidity is free from madness, as is supposed by one poet at least.

"Hail, awful Madness, hail !
Nor best nor wisest are exempt from thee.
Folly—Folly's only free."—*Penrose.*

Monomania is not incompatible with a high range of intellect on other subjects than the delusion. Simon Brown wrote his defence of the Christian religion—one of the best that was ever written—while labouring under insanity, and when he believed that his rational soul was dead, and that only his brute life remained. The remarkable fancies that have entered the imaginations of monomaniacs, would fill a thousand volumes. Perhaps the man described by Mr. Moore, the poet, who fancied he had been guillotined, and afterwards got a wrong head on his shoulders, is as ludicrous as any on record.

"Went to the mad-house,—saw the man
Who thinks, poor wretch, that while the fiend
Of discord here full riot ran
He like the rest was guillotined ;—
But that when under *Boney's* reign,
(A more discreet, though quite as strong one,)
The heads were all restored again,
He in the scramble got a *wrong one.*

Accordingly he still cries out
This strange head fits him most unpleasantly,
And always runs, poor devil, about
Inquiring for his own, incessantly !"

The above patient was in the Bicetre many years. Shakespeare, of all poets, has shewn, as it were from inspiration, the greatest knowledge of insanity. The more we read his works, the more we are astonished at the correctness of his portraits. His knowledge of the disease—its nature—and even its treatment, were far in advance of the age in which he lived.

" An examination of the writings will show that he believed the following facts, all of which were in advance of the general opinions of his age, and are now deemed correct.

" 1. That a well-formed brain, a good shaped head, is essential to a good mind.

" 2. That insanity is a disease of the brain.

" 3. That there is a general and partial insanity.

" 4. That it is a disease which can be cured by medical means.

" 5. That the causes are various, the most common of which he has particularly noticed.

" These assertions we shall endeavour to prove.

" *First.* That a well-formed brain is essential to a good mind, he often mentions. He particularly notices the excellence of a high forehead. Thus, Cleopatra, anxious to know the personal appearance of her rival Octavia, asks the messenger, 'Bearest thou her face in mind, is't long or round?' to which he replies, 'Round even to faultness, and her forehead as *low* as she would wish it.' This so pleased Cleopatra that she replied, 'There is gold for thee,' and rewarded him for his gratifying intelligence.

" So in the Two Gentlemen of Verona, Julia contemplating the picture of her rival Silvia, says, 'Her *forehead's low,* what should it be that he respects in her?'

" Again; Caliban, in the Tempest, fears they 'may all be turned to Barnacles, or to apes with foreheads villanous low!'

" *Second.* Shakspeare considered insanity to be a disease of the brain.

" In Macbeth, the struggle between sanity and insanity is well illustrated, particularly in the dagger scene. At first Macbeth doubts and asks:—

" Is this a dagger, which I see before me,
The handle towards my hand? Come, let me clutch thee."

" Not succeeding, he doubts his eye-sight and exclaims,

" Art thou but
*A dagger of the mind: a false creation,*
*Proceeding from the heat oppressed brain!*"

" Yet looking again, he sees it in form so 'palpable,' that he for an instant believes in its existence,—but finally reason triumphs and he exclaims,

" There's no such thing;
It is the bloody business, which informs
Thus to mine eyes."

" The whole passage is beautiful and instructive, and finely exhibits the struggle between reason and delusion.

" Macbeth also believed Lady Macbeth to be affected by mental disorder, and asks the doctor if he can not

" Minister to a mind diseased;
Pluck from the memory a rooted sorrow;
Raze out the written troubles of the *brain?*"

Showing that he considered her disorder seated in that organ."

Disordered mind is sometimes called by Shakespeare " brain-sickness." King Henry exclaims—" What madness rules in brain-sick men?"

The Poet of Nature believed that insanity was curable. Thus, in King Lear, Cordelia asks "What can man's wisdom do in restoring of his (her father's) reason?" The physician promptly and truly answers:—

"There are means, Madam;
Our foster-nurse of nature is repose,
The which he lacks, that to provoke in him,
Are many simples operative, whose power
Will close the eye of anguish."

Next to Shakespeare, Sir W. Scott has given the most exquisite delineations of insanity—more especially in the persons of Madge Wildfire—Norna, in the Pirate—and Clara Mowbray, in St. Ronan's Well. These are so well known that we need not notice them specifically.

We must now draw this short article to a close. In one of the articles of our new contemporary, there is a curious case of insanity, of which we shall exhibit a few particulars.

A gentleman, about 50 years of age, has been slightly deranged for twenty years past. His father was hypochondriacal, and he has a brother insane. He is a man of education, intelligence, and piety—of kind and amiable feelings and manners—conversing rationally on most subjects; but he is unable to walk, or to attend to any business requiring bodily exercise, without great mental agitation and ludicrous movements. He rarely stirs without much solicitation, and would remain in his room all day, if not compelled to move. He occasionally writes verses, and the following ode to the *Hypo* is not unamusing.

"No tongue can declare
The torment I bear,
It my heart-strings doth tear,
So keen are the pangs of the Hypo.

I start 'cross the floor,
Then pitch out the door,
As if ne'er to enter more,
In order to fly from the Hypo.

I then dodge and run,
Which often makes fun,
Till my race is quite done,
I am so bother'd by the Hypo.

I pick up a chip,
A stone or a whip,
And along hop and skip,
And this is to fool the Hypo.

And 'tis not in vain,
For my object I gain,
And I will not complain,
For hereby I master the Hypo.

I see people laugh,
Though they'd better cry by half,
But I then seize my staff
And rush to the combat with Hypo.

I could sit down and cry,
And pour floods from my eye,
And weep till I die,
I am so afflicted with the Hypo.

But this will not do
I plainly do know,
For it adds to my woe,
And only increases the Hypo.

No, I must resist,
As if fighting with fist,
And sometimes must twist,
Or soon I shall die with the Hypo."

It is consolatory to know that this poor victim to HYPPO was greatly relieved by a seton in the back of the neck—warm-baths and other remedies, so that he returned to his family, where he will probably favour us with another ode to HYPO—we sincerely hope a FAREWELL ODE.

We now take leave of our amusing contemporary, and, although his LOCUS PENITENTIÆ is "pent up in Utica," we trust that the products of his brain will have a wide range—trans and cis-Atlantic.

---

FURTHER OBSERVATIONS ON THE USE OF NITRIC ACID AS AN ESCHAROTIC IN VASCULAR TUMOURS OF THE RECTUM. By *John Houston*, M.D. Surgeon to the City of Dublin Hospital.

Since Dr. Houston's first Essay on this subject in our Dublin contemporary, he has had many additional opportunities of testing the efficacy of the application in question, and the results he considers to authorize a supplemental communication. The cases here published are selected wholly from hospital practice, as being more authentic documents than those furnished from the note-book of the private surgeon. Dr. H. reiterates his caution that the nitric acid is only applicable to the internal bleeding pile—" that soft, red, strawberry-like elevation of the mucous membrane,—for which I have used the term *vascular* tumour—and which the acid removes by the production of a slough of its surface."

" The surface to be thus acted upon must be soft, and free from any coating of cuticle, such as is apt to form on it by persistent prolapse; for, if the acid be used in a case so circumstanced, nothing more than a removal of the cuticle may be expected from the application; and further, to ensure to the caustic its full effect, the part to be touched by it should, beforehand, be dried and cleared of all mucous or other adherent fluids. There is no danger, that I know of, to be apprehended from the application of the acid; I have never seen any consequence from it beyond what I have stated in my reports. But, to be successful, the remedy must, of course, be used with decision. The acid must be laid on in quantity, and rubbed in with force, enough, to be pressed into the pores of the surface. At the best, it produces only a very superficial slough; and, on this account, it will be necessary in some cases, as where the tumours are old and firm in texture, to make a second, and even a third, application. Of this, the patient should, of course, be informed beforehand, that he may not be taken by surprise, in case any such necessity should arise."

The great value of this remedy lies in its applicability to general practice, and in the readiness with which invalids can be prevailed upon to submit to it. Dr. H. has detailed six or seven cases in his present paper, of which we shall here insert one as a specimen.

" *Case* VI.—William Gray, æt. 32, a tall man, a tide-waiter, temperate, and much exposed to wet and cold, admitted Feb. 12, 1844. He has laboured under his present indisposition for about five years, but during the first year he did not regard it much, as his only inconveniences were those of constipation; out of this arose a habit of taking aperients, which by producing tenesmus, long sittings at stool, pruritus ani, prolapse, and hæmorrhage, only made matters worse. About six months ago, he suffered a more than usually severe attack of this kind, with considerable prolapse, pain, and loss of blood, and from this period these symptoms did not, as formerly, go off; on the contrary, they became aggravated and accompanied in addition with muco-purulent discharges, which, in spite of his best efforts at cleanliness, kept his linen soiled. The part was touched with solutions of sulphate of copper, caustics, astringent tinctures, &c., and purgatives were administered, by different medical gentlemen, but such treatment only irritated, without diminishing the protrusion or lessening the discharges.

"*State on Admission.*—The muco-purulent discharges are now constant; the blood, occasional; he has almost continually pruritus; he finds difficulty in controlling his inclinations to sit long, and strain at the night-chair, after the evacuation is completed, notwithstanding his long experience of the fallacy of the sensation of there being still something to come away, and his conviction that he has the power of at once relieving it by returning the prolapsed part to its place. He has rarely a stool, unless as the result of medicine; the tongue is white, and he complains of thirst, bad appetite, and flatulence. He suffers from an uneasy sense of fullness, with a glow of heat, and pulsatory throbbing inside the anus.

"On straining in the sitting posture, a protrusion takes place, at least an inch and a half in length, spreading broad over the sides of the anus, and exhibiting three large, blood-red tumours on its mucous surface. The whole texture of the protruded part is tumid from congestion, but the *vascular tumours* stand out beyond the general surface of the prolapse, although they do not, to the feel, exhibit any greater density or hardness than the other parts. Blood oozes from them if they be allowed to remain protruded for any time; the prolapse is not at present at its worst, nor is it accompanied with pain; there is little appearance of varices in the neighbourhood, either inside or outside the anus.

"On the evening of the 13th I administered a purgative draught. On the 14th, while the prolapse was at its greatest projection, I applied the nitric acid effectively to all the tumours. The application caused them to bleed freely; but this was more from the friction on their turgid surface, than from the escharotic effects of the acid; oil was then applied, and the protrusion carefully reduced. The man was placed on his back in bed, with directions not to allow the prolapse to recur; an opiate was administered. The pain of the application was not much complained of.

"15th. He says that he suffered some pain, heat, and throbbing yesterday, after the touching, but that it was of a distressing kind; he was lulled from the opium, but did not sleep; he had, unexpectedly, a free motion during the night, unattended with any unusual pain, and occurring without prolapse. There is, this morning, a projecting, pale, œdematous ridge of the mucous membrane at the margin of the anus,—an evidence of inflammatory action, inside, induced by the irritation of the caustic.

"16th. Found him walking about, at the hour of visit; he feels no pain or discomfort whatsoever, except when he coughs or makes some bodily exertion, and then he is only reminded of his ailment by the part being hurt a little in the effort.

"18th. The bowels have been moved without medicine; the evacuation consisting of fæces stained with pus and sanies (the discharge from the cauterized surface), but not with unmixed blood; there was no prolapse with the movement, and the tumours did not come into sight; nothing is now to be seen wrong, and there is only a slight soreness on pressure.

"20th. He left the hospital sufficiently well to go about his ordinary business."

We need hardly say that this is one of those short, practical, and valuable papers which contain more information than some of the lean or dropsical octavos which we are too often destined to wade through.

TRANSACTIONS OF THE NEW YORK STATE MEDICAL SOCIETY. Vol. VI. Part I. Albany, 1844.

FROM a talented address to the New York State Medical Society, on the subject of insanity, we extract the following practical remarks :—

"In the therapeutical treatment of insanity, every case must be considered and treated as an insulated one. Remedies must be applied to the constitution and peculiar features of each case. While the first indication is to remove or lessen, as far as possible, irritation as the immediate cause, pervading the cerebral and nervous system, and through sympathy the vascular, yet are we to bear in mind the condition of other remote organs morbidly excited, and participating in the general disturbance. For instance, the associative powers of the stomach as a central organ are immensely important, as it regards the phenomena of disease. So also, through arterial agency, defective secretion of the gastric juice, and loss of power in the secerning system, we account for local congestion, impaired appetite, and waste in fevers.

"Remedial means when rightly applied need be but few. And what is the popular aim in the cure of diseases, at the present day? but to sustain the conservative principle, the strongest in nature, by the revulsion of excitement to parts less essential to life, and equalising circulation. Hence the importance that our first move in the treatment of incipient insanity, should be based upon a correct diagnosis; critically regarding the necessary distinction ever to be maintained between phrenitis, and active mania. The one concentrated inflammation, affecting the substance and meninges of the brain, the other, irritation specifically embracing the nerves of sensation and volition, sympathetically disturbing every function and fibre of the human system. The first demanding bold depletion as the anchor of safety, the latter to be approached cautiously, by milder and more comprehensive means, as we shall proceed to enumerate.

"Here then, permit me to remark that no one is competent to endure this searching ordeal who is not well versed, analytically and pathologically in every branch of medical science.

"Copious abstractions of blood should ever be avoided in insanity as endangering dementia. Very few are the cases of insanity, even in its incipient stage, that admit of venesection. In such only as are plethoric and in the vigor of life, is it admissible at all, and then only in a cautious degree. The pulse is deceptive, for though there may be increased impetus of blood in the carotids, yet they will be found compressible, and the radial artery feeble in its action, showing an unequal distribution rather than congestion. In such cases where symptoms seem urgent, topical bloodletting by leeching, or cupping, may safely be resorted to without danger of collapse. In the treatment of six hundred cases, venesection has not been resorted to in more than one in a hundred, after they entered the institution, and then only moderate in quantity. Many, however, have been brought to the Asylum, after two or three copious bleedings, undoubtedly with the best intentions; yet the results have proved a prostration of the vital energies, more difficult to overcome than the original disease.

"Puerperal insanity, which generally comes on within a week after delivery, arises from a metastasis of morbid lacteal and lochial secretions, is attended with rapid pulse and great prostration of strength. Hence bleeding is inadmissible. If prudently managed it seldom terminates in permanent insanity. The head and uterus principally suffer, tension and pain of the abdomen being less than in puerperal fever. Generally ushered in by sudden impressions of cold from a higher to lower temperature. Hence the retrocession, and the necessity of restoring the secretions.

"Active emetics are seldom admissible as tending to a determination to the brain. Where there is great derangement of the digestive organs, ipecac and calomel combined, in such quantities as to produce a slight emetico-cathartic effect, may prove salutary in their operation. So also the blue mass with one fourth part of ipecac, adds to its efficiency in restoring the functions of the liver.

"Drastic purges are seldom advisable. Laxatives to keep up a steady action of the intestinal tube are far preferable, and may be aided by injections, due exercise, and a well regulated diet. No particular formula can here be laid down. The judgment of the physician must decide on the quantity and appropriateness of the article, according to the constitution and peculiarities of the patient.

"Narcotics and sedatives are next in order. Opium, camphor, morphia, stramonium, conium, belladonna, and aconite are most to be relied upon, but require great prudence as to the time and manner of their administration. These are often improved by combination with other remedies. For instance, opium, ipecac, and soap, equal parts, forms a pill much easier given than Dover Powders. Camphor mixture, with half a grain of tart. antimony, and five drops of laudanum to the ounce, given in half-ounce doses, is a powerful sedative and adjuvant in allaying nervous excitement. Morphia with colchicum when there is a gouty or rheumatic diathesis, endangering metastasis, is a valuable auxiliary in treatment. Stramonium acts specifically on the sensorium, stimulating the absorbents. A saturated tincture of the seeds in camphor mixture, is the best mode of administering it. Conium is best combined with the different preparations of iron. Belladonna and aconite are often improved by combination. Extracts of these vegetables can only be relied upon when evaporated by solar heat.

"Counter-irritants, revulsive in their effects are valuable auxiliaries, more especially in metastasis and suppressed erruptions; and are more cheerfully submitted to when allayed with some of the vegetable narcotics endermically applied.

"Bathing. One of the most powerful remedial agents in equalizing circulation, is the warm bath. The patient should be immersed from twenty to thirty minutes, the heat at 96° Fahrenheit, refrigerating the head while in the bath, when the heat of the part should indicate its necessity. Warm bathing will be found particularly beneficial and appropriate in melancholia and delirium tremens. Fixed alkaline salts added to the water, are useful in removing the sebaceous oil from the surface of the body. The nitro-muriatic bath is a valuable and effective agent in a congestive state of the liver, and should be repeated in connection with the usual remedies, until we have evidence of a healthy secretion of bile. The value of the shower bath is known to all, yet it is too indiscriminately used. Great prudence and watchfulness is necessary in its application. Should atony prevent a suitable re-action and warmth over the surface, it may do serious and lasting injury, A pitcher of cold water poured over the back part of the head is often grateful as well as useful to the patient.

"In the second stage of insanity, a more tonic treatment becomes necessary, and it is to be regulated according to the age, constitution, and temperament of the patient. The various preparations of iron, mineral acids and quinine, nitrate of silver, followed with a solution of iodine to prevent a discolouration of the skin, conjoined with suitable moral treatment, will often decide the future prospects and destiny of the patient.

"In moral treatment, no more restraint should be used than is absolutely necessary for their own and others' safety. Chains are never necessary in an institution. You cannot chain the human mind, nor cure a patient in chains. I have never used one. Pinel is entitled to an enduring monument of fame, for having been the first who dared to break off the chains of the maniac, throw open his prison door, bring him forth to the light of heaven, and to the enjoyment of the essential elements in their purity."

### THE AURISCOPE.

Dr. Warden, the inventor of this ingenious and useful instrument, has related the following cases in the September Number of Dr. Cormack's Journal.

"*Case* 1.—A. H., a maidservant, aged 20. My attendance was required in consequence of acute abscess of the right labium obstructing the evacuation of urine, and attended with symptoms of general fever. This condition was speedily relieved by the ordinary means. It afterwards appeared that leucorrhœal discharge had existed for an uncertain time, and in consequence of the renewal of the abscess and suffering, a more particular examination was instituted, the result of which was the detection of pathological conditions not ordinarily recognised as marking such an affection, and the knowledge of which has not entered into the indications of treatment in such cases. In explanation it is to be observed, that by the employment of the speculum above described, not only do we throw every requisite supply of light upon the parts at the extremity of the tube, but every point of the entire walls of the cavity is made to display its picture upon the interior prism in all its reality of visible aspect, just in the same manner, and with the same truthfulness, as the pictures of the landscape in the *camera lucida*. By the use of this instrument in the case under notice, the os uteri was found turgid, rubescent, and giving issue to a profuse purulent discharge; and the examination of the walls of the vagina obtained through the medium of the second prism presented different diseased states, exemplifying a diversity of pathological conditions to which the mucous membrane is liable. In the neighbourhood of the os uteri, the inflammation was vesicular; in different portions of the parietes of the vagina there were patches of ulceration, of more and less vivid inflammation, and of warty vegetations, giving distinct indications for corresponding differences of topical treatment, and for which the speculum employed by me afforded all necessary facility upon the removal of the intervening glass tube. Within a fortnight these varied morbid conditions of the mucous surface have been treated with appropriate dressings, and a case which otherwise must have undergone an uncertain management and duration, will immediately admit of complete cure.

"*Case* 2.—Mr. G. S., aged 43, of bilious and leucophlegmatic habit, has suffered from impaired hearing, and disease in both ears, since his boyhood. The symptoms at the commencement are not distinctly remembered, but as deafness has been prevalent in his family, it is probable there was a morbid delicacy and predisposition affecting the organs in question. A marked and permanent aggravation of the dulness of hearing took place about 20 years ago, in the course of typhus fever. Constitutional and local treatment have been employed on various occasions, attended with slight and temporary improvement, but without preventing the progress of increasing loss of hearing. All remedies had been discontinued, as hopeless, for nearly two years, and hearing is produced only by bawling as to one on the house-top, the patient erecting the auricle with his hand to catch every vibration of sound. Upon examination with the auriscope the diseased condition of the affected organs gave little or no room to expect benefit from local or other treatment. The auditory passages were cleansed by washing with the hair pencil, when the cuticular lining was found blanched and thickened, as by maceration, and flocculent portions were readily separated from the surface, which was without the natural conditions of sensibility and ceruminous secretion. Upon carrying inspection to the situation of the membrana tympani, which is effected with facility by means of the prismatic auriscope, I found, in the right ear, that the bottom of the passage was occupied by a spongy organized mass protruding from the chamber of the tympanum, while, on the left side, a crescent-formed fragment of the drum only remained, and that having a lardaceous appearance, as if quite disorganized, and in progress of decay. In such unfavourable circumstances, I was chary of local interference, and advised a residence by the sea side, or in the country, while pursuing a course of mineral and bitter tonics, and general corroborating regimen. An astringent infusion was directed to be used as a wash for the external ears twice a-day, with attention to position in its employment, so as to avoid its penetrating to, and prematurely stimulating the diseased and feebly organized parts. After the lapse of a fortnight, the patient reported his health much improved, and his hearing also bettered. A weak zinc lotion and ointment were employed during another interval of a fortnight, and yesterday, the 12th instant, I had opportunity of examining the ears, when interesting changes of their condition were displayed by the auriscope,—and at same time the patient expressed high satisfaction that the day before he had been able to join in the psalmody of the church, which he had previously been incapable of, such was the deficiency of hearing. The external passages of both ears, which at first had a general sodden and oozing surface, were now almost free from moisture, compact and regular in texture, and some ceruminous secretion showed itself in the right ear. The fungus seen at first examination, had shrunk and disappeared, and a pretty uniform dusky structure filled the cavity of the tympanum. In the left ear, the relict of the membrane of the tympanum, formerly seen opaque and unorganized, had lost a little of its superficial area, but what remained had a healthy pinky vascularity and translucence like red jelly. Moreover, the white head of the malleus was seen in situ and in connection with this important fragment, although its handle stood in bold outline in the dark chamber of the tympanum.

"¶It is not to be expected that a case having such a history, and where essential parts of the auditory apparatus are deficient, should admit of any highly satisfactory issue in the way of cure. But it may be confidently anticipated that the same means which have been attended with such a measure of advantage, will be of yet greater service in process of time. A more vigilant inspection of the state of the parts affected will now be necessary, that the remedies adapted to their visibly more active condition may be employed, and their effects observed.

"*Case* 4.—I owe to your kindness my opportunity of seeing this and other such interesting cases under your charge in the Royal Infirmary. J. C. aged 20, a native of Sutherland, of strongly marked scrofulous constitution, has had a running from both his ears since he was eight years of age, attended with growing dulness of hearing: about 20 months ago the deafness became complete, after exposure to cold, which induced the symptoms of acute inflammation, with profuse suppuration, and the discharge of hard lumps from the ears. Since that time all his communications with others have been carried on in writing, a channel of intelligence of which happily he has ready command, as well as a power of expression beyond common. In his descriptions he essayed in no mean terms, *pingere sonum*—the rushing of the wind—the rustling of the leaves:—'and the murmuring main was heard, though scarcely heard to flow.' Having come to Edinburgh in the hope of recovering his lost hearing, he was fondly persuaded the impressions belonging to the sentient nerves were referrible to, and indicative of some feeble power in the proper auditory function. A watch, however, applied to the auricle and to the pericranium, or held between the teeth, gives no sense of hearing, neither do the loudest tones of the voice attract any attention; but what is still more conclusive of the entire abolition of that sense, his own voice is unperceived as giving sound, although he speaks with strong intonation. After washing the auditory passages, which were covered with an abundant slimy secretion, the view of their interior by means of the auriscope gave ample exhibition of the extent of the injury inflicted by continued disease. Not a vestige of either of the membrana tympani remained. The chamber of the right tympanum was partially occupied by pale granulations, which were seen immediately to become more vascular, and studded with bloody points on being lightly brushed with a hair pencil. The left tympanum was wholly empty, and its posterior wall was covered with a glairy pellicle, through which the *fenestra ovalis* was distinctly visible. An active seton had been some time in operation on the nape, and had relieved the head of oppression and other uneasy feelings; I therefore ventured on an attempt to alter the diseased surfaces, and to check the discharge, but action habitually morbid, manifested itself, in fungous granulation, and no benefit resulted. The case, contrasted with the former, serves to exemplify the ruling influence which the state of the general health and constitution exercises over diseases of the ear, and the remedies employed: and they are sufficient to argue the important aid to be derived from the prismatic auriscope, for revealing the features of disease, for dispelling the pernicious mystery in which the affections of these important organs have been hitherto invested, and for bringing them within the pale and protection of enlightened science.

" *Case* 5.—Mr. P., aged 45, has suffered from deafness for many years, resulting from chronic inflammation of the organs of hearing. In 1838, when hearing was all but completely lost, he repaired to Berlin to obtain advice of Dr. Kramer, and placed himself under his care for a considerable period. That eminent practitioner, according to his narrative of the case now before me, judged that the right ear was incurably deaf, owing to paralysis of the auditory nerve and other causes. The left ear alone, therefore, was subjected to treatment, in the opinion that the disease there consisted in and depended on stricture of the Eustachian tube, and an altered condition of the membranes therewith connected. According to this view, the measures employed were wholly aimed in one direction, namely to the quarter of the Eustachian tube: Dr. Kramer at same time noticing as his reason for abstaining from interference by the more accessible and important route of the external ear, that thickening of the membrane of the tympanum as a consequence of chronic inflammation, was an affection by no means curable. His efforts were directed to the dilatation of the stricture by a strong stream of air injected upon it by the air-press and catheter: afterwards the use of the catgut bougie was attenpted, but the irritation excited forbade its being persevered with. Through the daily use of the air-press, hearing was ultimately amended to the extent of the patient's hearing Dr. Kramer's watch at the distance of from one to two inches from the ear. This slight improvement was regarded as a great benefit at the time, and Mr. P. who has subsequently experienced a gradual amelioration of his hearing, is disposed to attribute this to the effect of the air-press as employed by Dr. Kramer. In expectation of more complete recovery, Mr. P. recently applied for my opinion of its probability, and the appearances presented by the auriscope enabled me to conclude that there was the fairest prospect of this in the remediable nature of the morbid state of the membrane of the tympanum. The appearances which I ascertained were these:—The lining of the auditory canal was healthy, but without ceruminous secretion. The membrane of the drum in about one half of its extent bore a perfect resemblance to the common integument of the body, being smooth, dense, and of uniform rosy vascularity; the remaining portion of the drum was of an opaque white colour, assuming a dark semi-translucency in its centre, not larger than a mustard seed. This darker spot had been mistaken by several observers for a perforation in the membrane, and this error had given occasion for a much less auspicious prognosis than the actual state of the case warranted. With the opportunity which is afforded by the prismatic auriscope of ascertaining the state of disease, and of watching and observing fully the effect of the remedies employed, I think that attempts may be safely made in this case to promote absorption in the opaque portions of the membrane, and to restore to a greater extent its natural elasticity and serviceableness for conveying the vibrations of sound.

" These few cases must serve in the mean time to illustrate the service afforded by the prism in revealing fully the characters of darkly seated disease,—a sequel to them I defer to another opportunity. I am, dear Sir, &c.

ADAM WARDEN, M.D."

